# Reduction of Waste Water in Erhai Lake Based on MIKE21 Hydrodynamic and Water Quality Model

**DOI:** 10.1155/2013/958506

**Published:** 2013-07-08

**Authors:** Changjun Zhu, Qinag Liang, Feng Yan, Wenlong Hao

**Affiliations:** ^1^College of Urban Construction, Hebei University of Engineering, Handan 056038, China; ^2^State Key Laboratory of Hydrology-Water Resources and Hydraulic Engineering, Nanjing Hydraulic Research Institute, Nanjing 210029, China; ^3^Nanjing Branch, Jiangsu Province Hydrology and Water Resources Investigation Bureau, Nanjing 210008, China

## Abstract

In order to study the ecological water environment in Erhai Lake, different monitoring sections were set to research the change of hydrodynamics and water quality. According to the measured data, MIKE21 Ecolab, the water quality simulation software developed by DHI, is applied to simulate the water quality in Erhai Lake. The hydrodynamics model coupled with water quality is established by MIKE21FM software to simulate the current situation of Erhai Lake. Then through the comparison with the monitoring data, the model parameters are calibrated and the simulation results are verified. Based on this, water quality is simulated by the two-dimensional hydrodynamics and water quality coupled model. The results indicate that the level of water quality in the north and south of lake is level III, while in the center of lake, the water quality is level II. Finally, the water environment capacity and total emmision reduction of pollutants are filtered to give some guidance for the water resources management and effective utilization in the Erhai Lake.

## 1. Introduction 

With the development of economy and natural science research capacities, the water environment and river health have drawn more and more attention [1]. MIKE21 is a two-dimensional mathematical model developed by DHI Water & Environment, which can be used to simulate water flow, waves, water quality, and sediment in rivers, lakes, seas, and bays. Water quality module (ECOLab) can be used for water quality simulation, forecast of water quality, water environment impact assessment, restoration of water environment and water environment planning, and so on. MIKE21 model has been used widely in the hydrodynamic, water quality and eutrophication. Lopes et al. studied the Ria de Aveiro Lake using MIKE21 model and proved that MIKE21 can be an effective tool to analyze the ecological system [2]. Wang et al. simulated the water flow and water quality of Jincang Lake in different designs, which provides scientific basis for ecological planning [3]. Chen and Wang studied the reconstruction project of Changxing Island and simulated and predicted the sediment using MIKE21 [4]. Liu and Yang studied the water pollution and eutrophication in Hanjiang river and Raihu lakeusing Mike model [5]. Wang et al. simulated the influences of drainage in Laibin on water environment of downstream using finite volume method by triangular/quadrilateral mixed grid [6]. Liang et al. simulated the water level of Hongze lake [7]; Yu et al. established a numerical model of diversion project of East Lake using MIKE21 [8].

Erhai is the second largest freshwater lake, which has important influence on people's life in Yunnan. It appears very important to control total amount of pollutants, which is not easy to directly control the quantity of pollutants in Erhai. According to the pollution sources and the actual situation of Erhai Lake, it is found that the pollution comes from the subrivers flowing into Erhai. As long as there is a good control of river water pollutant capacity, the total amount of pollutants in Erhai can be controlled. Share ratio method can clearly reflect the total amount of pollutants into lake bared by subrivers. 

In this paper, MIKE21 is used to simulate the water flow and quality in Erhai Lake, and the water environment capacity is calculated, which has reference for the water quality prediction and water quality control.

## 2. Methodology

### 2.1. Two-Dimensional Water Environment Model

The water environment model is composed of the hydrodynamic model and the advection dispersion model. The control equations can be written as

(1) $$\begin{array}{c} \frac{\partial \xi }{\partial t}+\frac{\partial p}{\partial x}+\frac{\partial q}{\partial y}=\frac{\partial d}{\partial t}, \end{array}$$

(2) ∂p∂t+∂∂x(p2h)+∂∂y(pqh)+gh∂ξ∂x+gpp2+q2C2·h2 −1ρw[∂∂x(hτxx)+∂∂y(hτxy)]−Ωq−fVVx +hρw∂∂x(pa)=0,

(3) ∂p∂t+∂∂y(q2h)+∂∂x(pqh)+gh∂ξ∂y+gqp2+q2C2·h2 −1ρw[∂∂y(hτyy)+∂∂x(hτxy)]−Ωq −fVVy+hρw∂∂y(pa)=0,

(4) ∂(hC)∂t+∂(uhC)∂x+∂(vhC)∂y =∂∂y(Exh∂C∂X)+∂∂y(Eyh∂C∂y)+S+F(C).

Equation (1) is the continuity equation, (2) and (3) are the momentum equations in the *x* and *y* directions, respectively, and (4) is the advection-dispersion equation. 

In the equations, *h*(*x*,  *y*,  *t*) is the water depth; *ξ*(*x*, *y*, *t*) is free surface water; *p* and *q*(*x*,  *y*,  *t*) are the discharge per unit width, m^3^/s/m; *g* is acceleration of gravity; *C*(*x*,  *y*) is Chezy resistance coefficient; *f* is the wind friction coefficient; *V* is the velocity; *Vx*, *Vy*(*x*,  *y*,  *t*) are the velocity in the *x* and *y* directions, respectively; *Ω*(*x*,  *y*) is the coefficient of Coriolis force; *C* is the pollutant concentration; *E*_*x*_ and *E*_*y*_ are the transverse and longitudinal diffusion coefficients, respectively.

These partial differential equations cannot be analytically solved, and a lot of mathematical solution methods have been developed, such as the finite difference method [9], finite volume method [10], finite element method [11], and finite analytic method. We take advantage of the simulation software MIKE 21 AD, which was developed by the Danish Hydraulic Institute with the finite volume method (Euler schedule), to solve these equations. It must be pointed out that the Courant number should be less than 1.0 in order to ensure the stability of the model (DHI 2005).

### 2.2. Water Environment Capacity

On the basis of investigation of pollution sources and monitoring of water quality, the numerical simulation is adopted to build hydraulic and water quality model. Response coefficient and share ration of various sources of pollution can be calculated. According to the water quality targets and the concentration, water environment capacity of controlled unit can be calculated.

#### 2.2.1. Response Factor Field

By analysis of water quality of lake and pollution sources, the corresponding relation is established between emission sources and the receiving water, which is the key of total pollutant. Concentration field (*C*_*i*_) formed by a strong source can be considered that it is composed of a plurality of unit source, namely the relationship can be established as follows:

(5) $$\begin{array}{c} \alpha_{i}=\frac{Q_{i}}{C_{i}}, \end{array}$$

where *α*_*i*_ is response coefficient which reflects the relationship of water quality with a certain point source. Obviously, because of the interactions among variety of environment power factors, the value of *α*_*i*_ changes with the location to form the response coefficient field. Response coefficient is the quantitative relationship between water quality in lake and pollution source on the basis of the mass conservation principle, which is the fundamental for the calculation of water environment capacity. Response coefficient field reflects the spatial features caused by rivers into lake. The highest value lies near mouth of the rivers into lake; along with the increase of distance, the influence of rivers flowing into lake is waning. Distribution situation of response coefficient is decided by river mouth position which is affected by environmental dynamic factors.

#### 2.2.2. Calculation of Share Ratio

**Figure 1:**
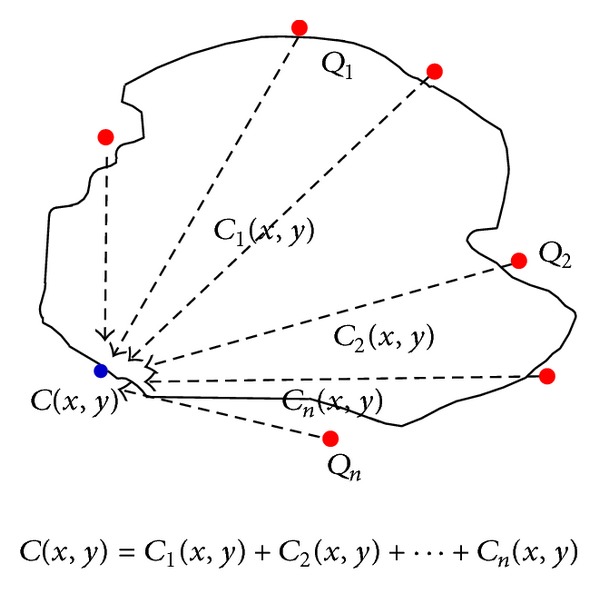
Stacking chart of concentration field.

According to the principle of linear superposition, the concentration field under the action of *n* pollution sources can be regarded as linear superposition of every single source which can be seen in Figure 1:

(6) $$\begin{array}{c} C\left(x,y\right)=\sum_{i=1}^{n}C_{i}\left(x,y\right), \end{array}$$

where *C*_*i*_(*x*, *y*) is the concentration field influenced by *i*th pollution source *Q*_*i*_(*x*, *y*) and (*x*, *y*) is space coordinate. Share ratio is the shares (%) influenced by every pollution source:

(7) $$\begin{array}{c} r_{i}=\frac{C_{i}\left(x,y\right)}{C\left(x,y\right)}. \end{array}$$

Share ratio indicates the degree of water pollution source to body water. Obviously, share ratio has the following characteristics: share is different in different regions at the same pollution source. A different pollution source has different share ratio.

#### 2.2.3. Emissions Quantity

According to the water quality standard *C*_0_(*x*, *y*), share ratio of concentration can be calculated under a different water quality target *C*_0*i*_(*x*, *y*):

(8) $$\begin{array}{c} C_{0i}\left(x,y\right)=r_{i}\cdot C_{0}\left(x,y\right). \end{array}$$

By sharing concentration, the further calculation of emission intensity can be calculated:

(9) $$\begin{array}{c} Q_{0i}=\frac{C_{0i}\left(x,y\right)}{\alpha_{i}}=\frac{r_{i}C_{0}\left(x,y\right)}{\alpha_{i}}. \end{array}$$

## 3. Case Study

### 3.1. Study Area

Erhai river basin is located in the watershed area of Jinsha river, Lancang river, and Yuanjiang river, which belongs to the Lancang-Mekong river. Watershed area is 2565 km^2^, and geographic coordinates are located in east longitude 100°05′~100°17′, and north latitude is 25°36′~25°58′. Erhai basin is located in Bai autonomous prefecture of Dali city in Yunnan province including 9 townships in Dali city and 8 townships in Eryuan country town and the Dali Provincial Economic Development Zone and Dali Provincial Tourism Resort.

### 3.2. Mesh Grid

The study area is an 20 Km width in east-west directions and 42 km long in south-north directions. It was meshed with square cells, where the maximum size of square cells was 400 m × 400 m. The number of modules of calculation is 50 × 105.

### 3.3. Conditions and Parameters

#### 3.3.1. Conditions

*( 1) Boundary Conditions*. There are 5 kinds of boundary (1) river into lake, (2) outlet, (3) drain outlet into lake, (4) water pumping station of industrial and agricultural, and (5) irrigation return water and rainfall and evaporation. Rivers into lake and drain outlet into lake can be recognized as pollution source, outlet and water pumping station of industrial and agricultural as sink, and irrigation return water and rainfall and evaporation as source. That is to say, source will be added into the continuity equation and the mass conservation equation of pollutants.

**Figure 2:**
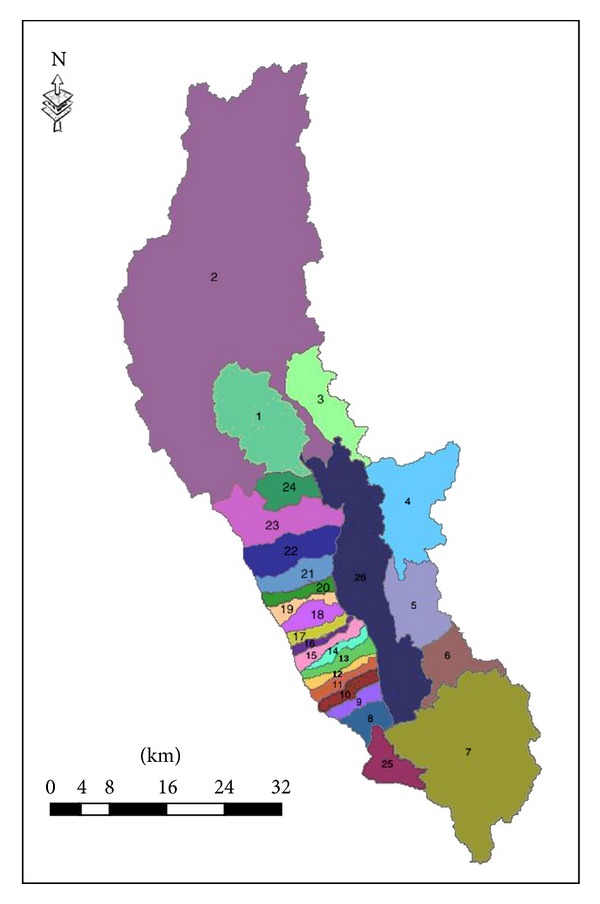
Rivers into the Erhai Lake.

According to the actual situation of Erhai, the pollution sources into the lake are incorporated into the Erhai river, and the Erhai river can be divided into 24 rivers shown in Table 1. The correspondence between 24 rivers into lake and watershed is shown in Figure 2.

*( 2) Selection of Feature Level*. The water level of Lake is an important factor for water environment capacity. In order to calculate the water environment capacity in Erhai Lake, the feature water level is selected as one of the basic conditions. In history, the minimum operation water level is 1971 m and the maximum water level is 1974 m. At the same time, according to the average monthly level from 1997 to 2008, the average operating level in recent 12 years is about 1973 m. Therefore, the feature level is selected as 1973 m (coastal evaluation).

*( 3) Initial Conditions*. The initial water level and water quality of lake can be given according to the calculated initial water level and concentration. The initial velocity of flow field is set to zero. Water environment capacity is also influenced by water quantity into lake in addition to the operating water level. Water level and water quantity should be considered when the water environment capacity in Erhai is calculated. According to the water quantity into lake for 54 years from 1956 to 2009, the P-III curve is adopted to calculate the experience cumulative frequency by test method. The arithmetic mean value, coefficient of variation (*C*_*v*_), and coefficient of variation (*C*_*s*_) are, respectively, 8.69, 0.30 and 0.45 billion square. According to the design frequency, during the study period 2000–2009, 2001 is chosen as the wet year (10.2 billion square), 2004 as mean year (8.9 billion square), and 2009 as dry year (5.7 billion square).

SWAT is adopted to be as the nonpoint source model. The water quantities into lake in wet, mean, and dry year are considered. According to the space relations, industrial, urban sewage, tourism, atmospheric deposition, surface source, and other various types of sources are counted. 

#### 3.3.2. Parameters

(*1) Hydraulic Parameters*

*(a) Roughness*. According to the characteristics of lake bed and lakeside, roughness can be set and adjusted for the calibration to get the roughness value which is 44. 

*(b) Dry and Flood*. Water level between water and land boundary can be determined by dry and flood. If the water level calculated has good fitting with the observation which will be involved in the calculation, if not, it will exit from the calculation. The general setting range of drying is 0.1-0.2 m and setting range of flooding is 0.2–0.4 m, and the difference of the two values is 0.1 at least. When the water level changes are fast with respect to the time step, the difference can be set to 0.2 or more. In this paper, drying depth is set to 0.2 m and flood depth is set to 0.3 m. 

*(c) Wind Resistance Coefficient*. Wind friction coefficient is a weak function of wind speed. For the medium and strong winds in open sea, wind resistance coefficient is adopted as 0.0026 to get good results. But to the breeze, a smaller coefficient is needed. If the changes of wind speed are set, the friction coefficient is set to be a change coefficient. The average speed of wind of Erhai is 4.1 m/s, so the friction coefficient is set to 0.0026. 

(*2) Water Quality Parameters*

*(a) Water Quality Parameters of COD*. Degradation coefficient *K*_COD_ of COD can reference the results of similar lake and are calibrated to 0.001/d according to the water quality simulation in 2009. 

*(b) Parameters on Total Nitrogen*. Degradation coefficient *K*_TN  _ of total nitrogen can reference the results of similar lake and are calibrated to 0.002/d according to the water quality simulation in 2009. Release rate of nitrogen in sediment *S*_N_ and the deposition rate of *K*_N_ were determined according to the release test of submarine mud. The value of *S*_N_ is 36.7 mg/(m^2^ · d), and the value of *K*_N_ is 30.9 mg/(m^2^ · d). 

*(c) Parameters on TP*. Degradation coefficient *K*_TP_ of total phosphorus can reference the results of similar lake and are calibrated to 0.004/d according to the water quality simulation in 2009. rate of settling of *K*_P_ and the release rate of *S*_P_ were determined according to the experiment. The value of *K*_P_ is 1.86 mg/(m^2^ · d), and the value of *S*_P_ is 1.13 mg/(m^2^ · d). 

### 3.4. Simulation of Water Quality in Erhai Lake

#### 3.4.1. Hydrodynamic Simulation

*( 1) Analysis of Flow Field*. Flows in Erhai Lake are influenced by interaction of wind-driven current and throughput flow and the wind-driven current is primary. Due to local topography, the wind style is complex. So wind-driven current in Erhai Lake is relatively complex.

**Figure 3:**
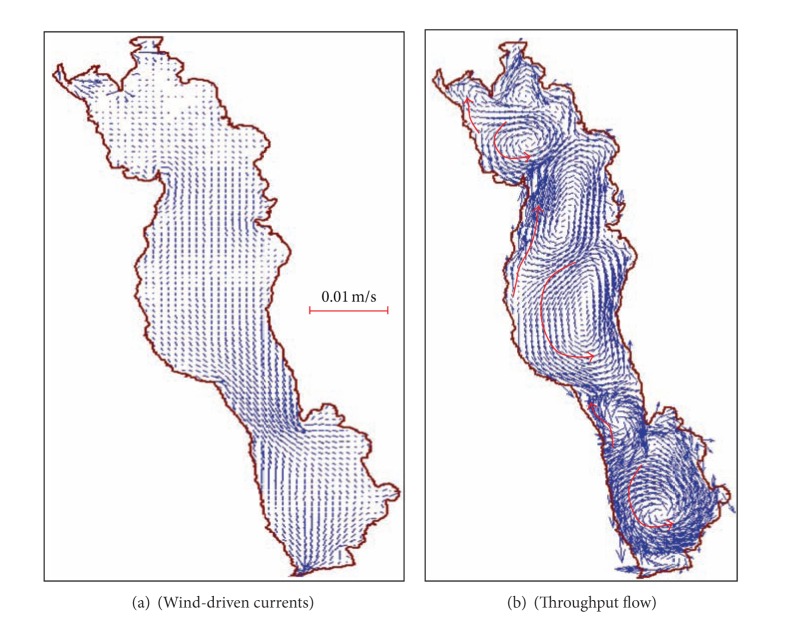
Wind-driven currents and throughput flow simulation diagram in Erhai Lake.

Figure 3(a) is flow state in Erhai Lake under the constant southwest wind driven. Flow in Erhai is affected by the southwest wind. And there is circulation in the north, center, and south of lake, and because of the influence of coastline shape and local topography, there is some small circulations outside the main lake. Under the influence of southwest wind with 4.1 m/s wind speed, the average velocity of lake current is 5.52 cm/s, and the ratio of wind speed and the velocity of lake current is 1.1%.

The typical patterns of throughput flow in Erhai Lake are shown in Figure 3(b). The lake flows with the throughput flow to downstream and there is no obvious cyclic lake flow. Because of larger flow in the north of the lake, the radiation flow is clear. Lake flow is in polymeric form in the south of lake. 

**Figure 4:**
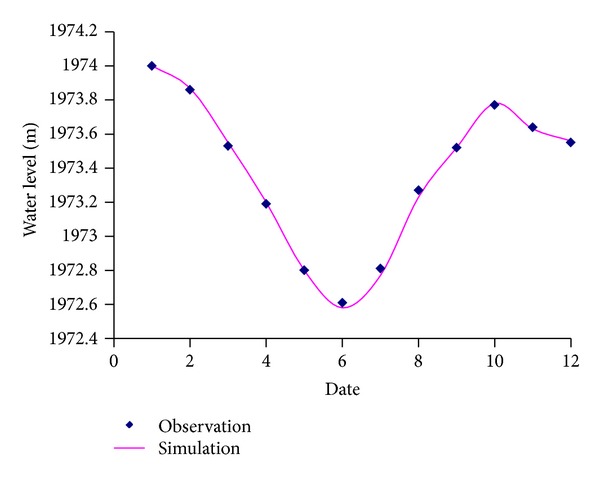
The contrast diagram of simulation value and measured value.

*( 2) Simulation of Water Level*. According to the meteorological conditions and water supply process in 2009, the simulation of Erhai Lake is developed during 2009 as shown in Figure 4. Seen from Figure 4, the simulated data and the observation data have good fitting. 

**Figure 5:**
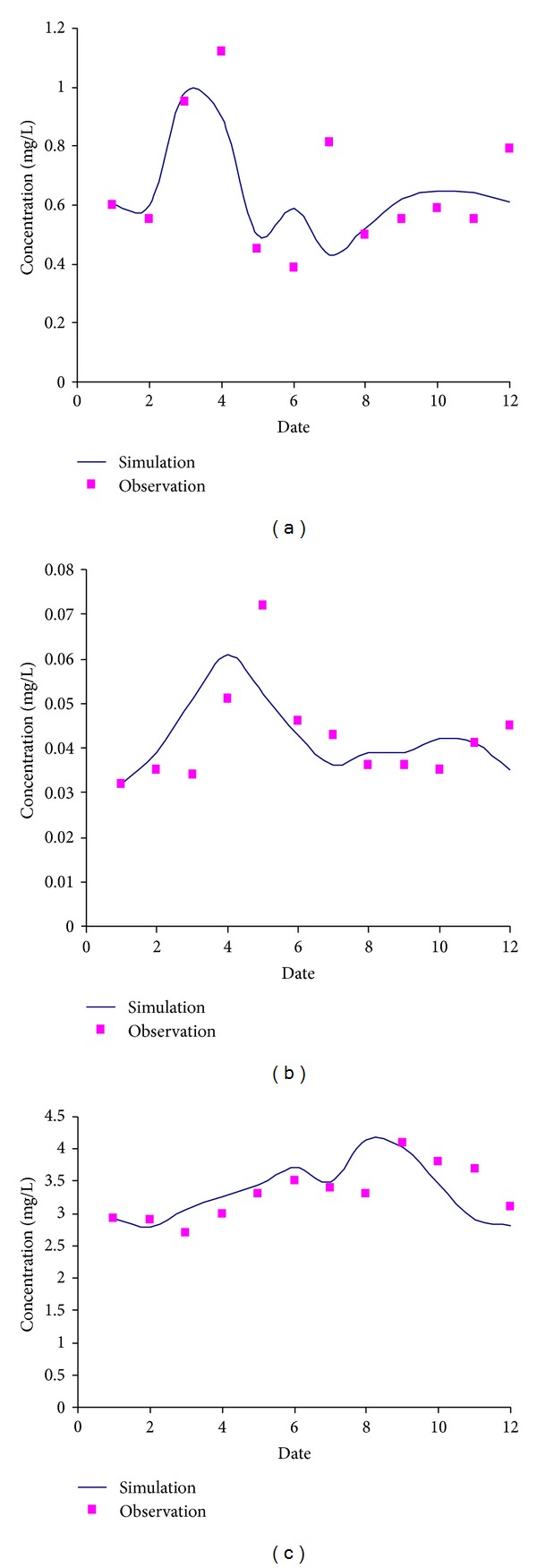
Comparison of simulation with observation in Daguanyi station.

*( 3) Simulation of Water Quality*. The two monitoring stations of water quality which are Daguanyi and Tuanshan stations are selected as the study object to verify the water quality model as shown in Figure 5. In all, the simulated results agree well with the observated data.

The concentration of all the Erhai Lake is between 0.48–0.78 mg/L, average is 0.60 mg/L, and in addition to March, April and December, TN values belong to level III. The concentration of TP is in the range of 0.01–0.037 mg/L, and average is 0.023 mg/L. TP values are level II from January to May, and November and December, while from June to October, TP was significantly increased to be level III. Permanganate index is in the range of 2.36–2.94 mg/L, and average is 2.58 mg/L which belongs to level II.

Seen from Figure 5, the relative errors of TN and TP are high, which may be due to the large amounts of pollutants of TN and TP or may be caused by monitoring errors. In all, the annual relative errors of TN are about 30%, while the annual relative errors of TP are about 30% also. The annual relative errors of COD are 10%. These all indicate that the simulation has high precision and the selection of parameters is reasonable.

#### 3.4.2. Analysis of Concentration Field

According to the simulation results of the water quality in Erhai Lake, distributions of the concentration on TN, TP, and CODMn are analyzed. The results indicate the water quality distribution in Erhai with the worst water quality in the northern Lake District, which is followed by the southern, and then the best in the central. The level of water quality in the north and south of lake is level III, while in the center of lake, the water quality is level II.

#### 3.4.3. Water Environmental Capacity

Selecting the monthly mean level from 1997 to 2008, the average is 1973 m. Using partition staging thought, water environment capacity in different regions at different times can be calculated to achieve the total water environment capacity. Staging is during the study period 2000–2009, 2001 is as the wet year, 2004 is as normal year, and 2009 is as dry year. Because Erhai is affected by the surrounding mountains terrain, the domain wind in many years and cangshan creek awallow spit flow to form the northern, central and southern three main circulation, erhai Lake district is divided into norhtern, central and southern lake.

Using numerical simulation, according to the observation and simulation, the concentration at any point can be got, and water environment capacity can be calculated according to the controlled point. This paper selects the water level under 1973 meters, central control in 2004. The relationships of response coefficient and share ratio of every river into lake can be seen in Table 2.

It can be seen in Table 2, to one river flowing into the lake, response coefficient and share ratio of three kinds of pollutants have a similar distribution; to different district, pollution influence of the same river flowing into lake is different. The closer to the lake it is, the higher the share ratio is. On the other hand, even near the mouth of lake, pollution effects are not completely caused by the river flowing into lake. Due to a large amount water into lake from Miju river, water environmental capacity coresponding Miju river is the maximum.

According to the share ratio and response coefficients of various rivers, water environment capacity beard by rivers flowing into lake can be calculated when the water quality in river meets various qualities. Water environment capacity of water reached Grade II can be further calculated which can be seen in Table 3.

By contrast, in recent years, the measured concentration of COD is higher than level II water standard. While the measured concentrations of TN and TP sometimes are level III. In littoral water of greatly human activities of North and South Baltic rivers into hukou, concentrations of TN and TP reached level IV water quality standard. Compared with the pollution load, pollution load of COD is no more than water environmental capacity below level II water quality, while TN and TP are more than water environmental capacity under level II water quality in some years matching the water quality situations, which indicate that the calculated water environmental capacity is reasonable. 

**Figure 6:**
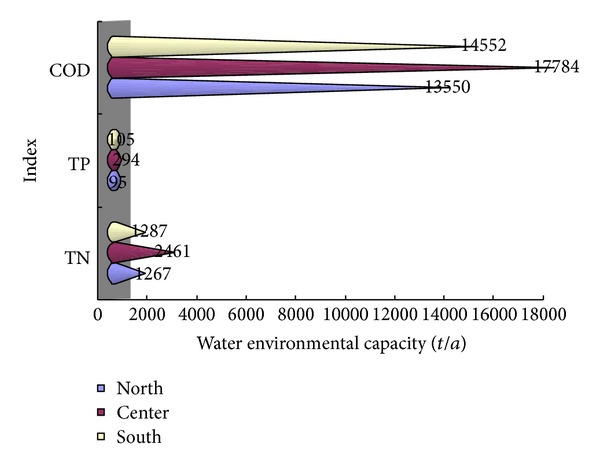
Water environmental capacity of mean year in level II water quality.

Water environmental capacity in the northern, central, and southern lake is calculated. Because there is differences in the water quality concentration in different zones, water environmental capacity is different under the control conditions.  Figure 6 is the water environmental capacity of main pollutants in the northern, central, and southern lake in the same year. Seen from Figure 6, water environmental capacity in central is the largest, followed by the southern, then the minimum in the northern. Inhomogeneity of distribution of concentrations of pollutants is the main reason causing the difference of water environmental capacity. Large amounts of pollutants in Miju river, Yonganjiang, and Luo shijiang the third longest river, which are in the north of lake, flow into the northern to accumulate the pollutants resulting the worst water quality. While there are less pollutants in the central, so the water quality is better and the water environmental capacity in central lake is larger. 

Given the water quality distribution of Erhai with the worst water quality in the northern Lake District, which is followed by the southern, and then the best in the central, there must have been some differences in the water environmental capacity calculated in the northern, central and southern control points. Because of poor water quality in northern lake, if water qualities in all the lake are ensured to meet standard, water environmental capacity calculated in northern controlled points is suggested to reduce the pollution into lake.

#### 3.4.4. Total Emissions of Waste Water

When the pollution load into lake is greater than water environmental capacity under a certain water quality objectives, water quality objectives meeting lake requirements cannot be achieved. Therefore, to ensure water quality objectives, pollution loads beyond the environment capacity should be reduced. The calculation is

(10) $$\begin{array}{c} \Delta Q_{i}=Q_{i}-{Q_{0}}_{i}, \end{array}$$

where Δ*Q*_*i*_ is the reduction of *i*th pollution load, *Q*_*i*_ is pollution emissions, and *Q*_0__*i*_ is water environmental capacity of *i*th pollution source. If Δ*Q*_*i*_ > 0, it shows that pollution load has exceeded the water environmental capacity and pollution load needs to be reduced. If Δ*Q*_*i*_ < 0, pollution load needs not to be reduced. Δ*Q*_*i*_/*Q*_*i*_ is reduction ratio. 

According to the standard of water quality protection to reach level II water quality standards, reduction load can be seen in Table 4.

## 4. Conclusions

Water quality simulation is one of the most important works for water resources protection and is an important part of digital river system, while digital river system is the future trend of water resources and management. With the help of MIKE21 model of water environment platform, water environment capacity in Erhai Lake was simulated. This paper aims to analyze the water pollution that will probably occur along Erhai Lake. This research could be used as a reference for forecast and protection of water pollution in Erhai Lake. The following conclusions can be drawn.

1. The numerical model, based on the software of MIKE21, has the capacity of simulating the water level and water quality distribution. This model could be used for forecasting and numerical experiments for further research.
2. On the basis of water quality-water dynamics model, pollution load, and hydrometeorological characteristics of Erhai, water environment carrying capacity of three typical hydrological period (dry, normal, wet) has been calculated. If water quality of Erhai has achieved level II, share ration can clearly show the proportion of each river flowing into the lake, which can be reached from control of the water quality of all rivers to the control objectives purpose. According to the pollution load of river itself, the reduction of pollutants can be calculated to study how the pollutants are abated.

## Figures and Tables

**Table 1:** Main rivers into Erhai Lake in Erhai basin.

Number	The river name
1	Luoshijiang
2	Miju river
3	Yonganjiang
4	Fengweixi
5	Haidongjing
6	longwangmiaojiang
7	Boluojiang
8	Yangnanxi
9	Tingqixi
10	Mocanxi
11	Qingbixi
12	Longxi
13	Baihexi
14	Zhonghexi
15	Taomeixi
16	Yinxianxi
17	Shuangyuanxi
18	Baishixi
19	Lingquanxi
20	Jinxi
21	Mangyongxi
22	Yangxi
23	Wanhuaxi
24	Xiayixi

**Table 2:** The relations between response coefficient and share ratio.

Number	Share ratio/response coefficient		
	TN	TP	COD
1	152.67	170.40	256.04
2	644.20	1274.84	1207.72
3	99.75	64.24	156.03
4	149.03	188.75	154.09
5	54.30	93.81	87.88
6	101.76	58.35	67.30
7	504.04	696.32	388.37
8	153.37	172.84	115.65
9	23.63	47.15	35.06
10	33.78	74.23	49.80
11	27.78	68.31	23.70
12	44.04	63.72	44.31
13	83.81	133.81	154.93
14	57.62	84.88	91.30
15	50.62	91.21	54.09
16	26.13	39.54	14.64
17	31.80	37.89	26.03
18	42.13	41.97	40.13
19	23.99	36.16	23.23
20	28.18	66.41	32.25
21	28.75	56.04	64.37
22	59.73	99.80	92.91
23	76.91	164.43	138.52
24	36.66	77.07	69.12

**Table 3:** The calculations of water environmental capacity in Erhai Lake (*t*/*a*).

Period	Control point	TN			TP			CODcr		
		I	II	III	I	II	III	I	II	III
Wet	North	527	1318	2636	39	98	195	7798	15596	23394
Normal	North	507	1267	2535	38	95	191	6775	13550	20325
Dry	North	431	1077	2155	36	89	179	5385	10770	16154
Wet	Center	1065	2661	5323	129	321	643	10479	20958	31437
Normal	Center	984	2461	4922	118	294	588	8892	17784	26676
Dry	Center	843	2107	4213	108	270	539	7275	14549	21824
Wet	South	543	1358	2715	44	109	218	8059	16118	24177
Normal	South	515	1287	2574	42	105	211	7276	14552	21828
Dry	South	462	1156	2311	37	93	186	5847	11694	17541

**Table 4:** The reductions of pollution load in Erhai Lake *t*/*a*.

Hydrological period number	Wet period			Normal period			Dry period		
	TN	TP	CODcr	TN	TP	CODcr	TN	TP	CODcr
1	70.74	3.64	—	44.43	1.67	—	16.74	0.75	39.65
2	317.37	27.24	—	187.45	17.88	—	92.69	5.23	186.02
3	39.75	1.37	—	29.02	0.74	—	13.32	0.33	24.38
4	54.01	4.03	—	43.36	2.09	—	17.01	1.02	23.77
5	20.64	2.00	—	15.80	1.26	—	7.54	0.62	13.54
6	36.37	1.25	—	29.61	1.51	—	10.32	0.60	10.35
7	177.96	14.88	—	146.67	9.69	—	52.09	4.02	59.90
8	54.04	3.69	—	44.62	3.15	—	31.39	1.65	18.09
9	9.19	1.01	—	6.87	0.95	—	4.72	0.48	5.39
10	13.33	1.58	—	9.83	1.04	—	6.68	0.49	7.67
11	10.45	1.46	—	8.08	1.09	—	5.65	0.49	3.65
12	16.02	1.36	—	12.81	1.67	—	8.55	0.70	6.84
13	30.13	2.85	—	24.38	2.48	—	17.11	1.25	23.95
14	21.44	1.82	—	16.77	2.02	—	11.74	0.95	14.15
15	18.36	1.95	—	14.73	1.61	—	10.45	0.73	8.35
16	10.38	0.84	—	7.60	0.49	—	4.87	0.25	2.26
17	12.93	0.81	—	9.26	1.06	—	6.41	0.48	4.01
18	17.69	0.90	—	12.26	1.09	—	7.54	0.49	6.18
19	9.42	0.78	—	6.98	0.62	—	4.51	0.30	3.58
20	12.36	1.42	—	8.20	0.93	—	5.52	0.43	4.97
21	11.36	1.20	—	8.37	0.82	—	5.60	0.44	9.91
22	27.59	2.13	—	17.38	1.14	—	11.27	0.65	14.30
23	32.39	3.51	—	22.38	2.76	—	14.95	1.32	21.43
24	15.13	1.64	—	10.67	1.20	—	7.43	0.65	10.71

Total	1039.04	83.38	—	737.90	59.32	—	374.07	24.32	523.05

“—” is Δ*Q*_*i*_ < 0, and the amount of pollutants into lake is less than water environmental capacity.
